# Personalized Psychological Interventions in Children: Harnessing Advancements in Genetic and Epigenetic Research

**DOI:** 10.3390/jcm12165311

**Published:** 2023-08-15

**Authors:** Luca Cerniglia

**Affiliations:** Faculty of Psychology, International Telematic University Uninettuno, Corso Vittorio Emanuele II, 39, 00186 Rome, Italy; luca.cerniglia@uninettunouniversity.net

Advancements in genetic and epigenetic research have opened new avenues for personalized psychological intervention in children. The knowledge that genetic and epigenetic factors significantly contribute to individual differences in emotional and behavioral responses has transformed the field of child psychology. This editorial explores the potential of using genetic and epigenetic information to tailor psychological interventions, thereby optimizing treatment outcomes and promoting children’s mental well-being. By combining cutting-edge genomic research with evidence-based therapeutic techniques, personalized psychological interventions hold promise in addressing the unique needs of each child.

Children’s psychological well-being plays a crucial role in their overall development and life outcomes. Traditionally, psychological interventions, for example, in the field of feeding and eating disorders [1,2,3], have adopted a one-size-fits-all approach, aiming to address common symptoms across various individuals. However, emerging research in the field of genetics and epigenetics has revealed that individual variations in emotional and behavioral responses are influenced by genetic predispositions and epigenetic modifications [4,5].

Genetic factors have been shown to contribute significantly to the development of various psychological traits in children, such as temperament, personality, and vulnerability to mental disorders. Additionally, epigenetic mechanisms, which regulate gene expression without altering the underlying DNA sequence, play a crucial role in the interplay between genetic predispositions and environmental influences. Understanding these complex interactions can help design tailored interventions that address the unique needs of each child.

Genomic profiling, which involves analyzing an individual’s genetic makeup, provides valuable insights into their predisposition to specific psychological traits and disorders. By identifying genetic markers associated with behavioral tendencies and psychological vulnerabilities, personalized psychological interventions can target specific areas of concern effectively.

Epigenetic modifications serve as a bridge between genetic predispositions and environmental experiences. Childhood experiences, such as trauma, neglect, or supportive caregiving, can induce epigenetic changes that influence emotional and behavioral responses [6,7,8,9]. Utilizing epigenetic information in personalized interventions can help tailor therapies to address the unique effects of early life experiences on the development of children.

With the integration of genetic and epigenetic information, therapists can design personalized interventions that align with a child’s specific needs and susceptibilities. Targeted therapeutic strategies can be developed to address genetic factors associated with risk or resilience to certain mental health conditions, while also considering epigenetic influences shaped by environmental factors [10].

While personalized psychological interventions offer great potential, ethical considerations must be prioritized. Informed consent is essential when incorporating genetic and epigenetic information into treatment plans, ensuring that parents and children fully understand the implications and potential benefits of personalized approaches [11].

Despite the immense potential of personalized psychological intervention, several challenges exist, including the complexity of genetic and epigenetic interactions, data privacy concerns, and the need for interdisciplinary collaboration. Further research is required to establish the efficacy and long-term effects of such interventions in diverse populations.

The integration of genetic and epigenetic information into personalized psychological intervention marks a significant advancement in child psychology [12]. Tailoring therapeutic approaches based on individual genetic and epigenetic characteristics offers an opportunity to optimize treatment outcomes and improve the mental well-being of children. As research in this field continues to evolve, it is imperative to strike a balance between scientific advancement, ethical considerations, and the best interests of the child, ultimately fostering a future where children can thrive with personalized psychological support [13].
